# Dual-Polarized Metasurface-Integrated Antenna for Integrated Imaging of LWIR Camera and SAR

**DOI:** 10.3390/mi16020202

**Published:** 2025-02-10

**Authors:** Jijian Hu, Zhenghong Dong, Lurui Xia, Xueqi Chen

**Affiliations:** School of Space Information, Space Engineering University, Beijing 101416, China; yuanfang202201@163.com (J.H.); xlrui522@hgd.edu.cn (L.X.); chenxueqi@hgd.edu.cn (X.C.)

**Keywords:** integrated imaging, metasurface-integrated antenna, dual-linearly polarization, dual-circularly polarized

## Abstract

The integrated imaging of LWIR cameras and SAR is one of the important directions of multi-sensor integration. In order to reduce the structural complexity of LWIR cameras and SAR-integrated imaging antenna, a dual-polarized metasurface-integrated antenna (MIA) is designed in this paper. It is composed of a microwave metasurface antenna and an optical metalens, and the metalens is embedded in the center of the metasurface antenna. The MIA uses the powerful electromagnetic wave control ability to simplify the optical and microwave signal transmission paths and reduce the number of devices. At the same time, in order to expand the function of the MIA, based on the principle of metasurface, the dual-linearly polarized and dual-circularly polarized MIAs are designed and simulated, respectively. The results show that the designed dual-polarized MIA has good performance. This paper provides a new scheme for the integrated imaging system of LWIR cameras and SAR with simple structure, diverse functions and easy integration.

## 1. Introduction

In recent years, a single remote sensing image is increasingly unable to meet the needs of applications. With the advancement of technology, the method of fusing multiple remote sensing images of the same object to obtain more information has played an important role in many fields [1,2,3,4]. Synthetic aperture radar (SAR) and LWIR cameras are two important remote sensing payloads. In order to acquire optical and SAR images of the same target at the same time, the integrated technology of LWIR cameras and SAR was born and have developed continuously [5,6].

At present, LWIR cameras and SAR-integrated imaging systems are mainly designed based on common aperture antennas. The system integrates the SAR antenna reflector and the primary mirror of the LWIR camera through common aperture antennas so that the primary mirror reflects microwave and optical signals at the same time. And the system designs different transmission paths for the microwave and optical signals to reduce mutual interference. According to the different LWIR camera structure, common aperture antennas can be divided into two types, coaxial [7,8,9] and off-axis [10]. The common aperture antenna has the advantages of high aperture utilization and long operating distance. However, the large volume, complex structure and many devices of the common aperture antenna are not conducive to the miniaturization and integration of the system.

Metasurface is composed of special subwavelength structures arranged according to specific laws, which has a strong ability to regulate the amplitude, phase and polarization of electromagnetic waves. The microwave antenna based on metasurface design has the advantages of low profile, multi-polarization and reconfiguration [11,12,13], and the optical metalens has the advantages of thin, diverse functions, and easy miniaturization and integration [14,15].

In order to reduce the structural complexity of the system, the dual-polarized metasurface-integrated antenna (MIA) is designed for integrated imaging of LWIR cameras and SAR. Firstly, the structure of dual-polarized MIA is introduced. Secondly, the principles of the microwave metasurface antenna and optical metalens are analyzed. Then, the units of the metasurface antenna and metalens are designed, respectively. Finally, the performance of the dual-linearly polarized and dual-circularly polarized MIA is analyzed by simulation, and the rationality of the design of the dual-polarized MIA is verified.

## 2. Materials and Methods

### 2.1. Dual-Polarized MIA Structure

**Figure 1:**
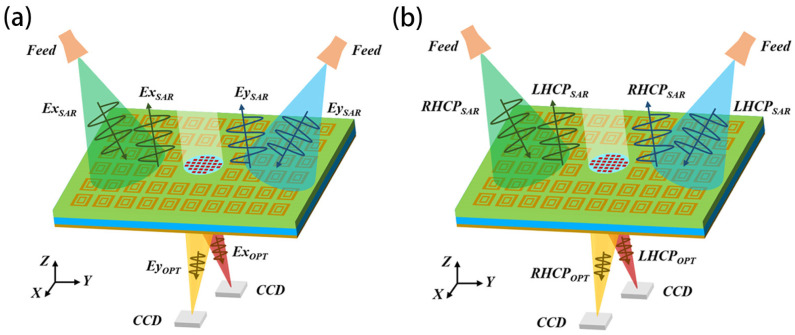
Schematic diagram of dual-polarized MIA (**a**) linear polarization; (**b**) circular polarization.

The dual-polarized MIA designed in this paper consists of a microwave metasurface antenna and an optical metalens, as shown in Figure 1. The metasurface antenna is a reflector array antenna, and the feed is a horn antenna. The metalens is embedded in the center of the metasurface antenna and is fixed using the non-metallic material whose dielectric constant is similar to that of the metasurface antenna substrate. The vertical axis of the metalens and the vertical axis of the metasurface antenna are combined in order to make the MIA image the same target simultaneously with optical and SAR. In addition, the light of the metalens is vertically incident, and the radiation direction of the metasurface antenna is the vertical axis direction; that is, the metasurface antenna and the metalens are focused in the same direction. After the microwave signal is sent out by the feed, it passes through the reflective array to form the required beam. The optical signal is focused on the CCD by the metalens.

The dual-linearly polarized metasurface antenna has two feeds excited by different linear polarization modes. The central coordinates of the feeds are located in the YOZ plane and are symmetrically distributed on the Z axis. The microwave signals emitted by two feeds both can be controlled by the reflector to form the desired beam. The dual-linearly polarized metalens focuses the X and Y polarized light on the CCD, respectively. In order to reduce interference between the microwave signal and the optical signal, the focuses are set on the XOZ plane and are symmetrically distributed on the Z axis. The structure of dual-circularly polarized MIA is the same as the dual-linearly polarized MIA; the difference is their polarization mode.

Compared with the common aperture antenna, the dual-polarized MIA simplifies the transmission paths of microwave and optical signals, reduces the number of devices, has simpler structure and is conducive to integration. At the same time, both the metasurface antenna and the metalens realize the independent regulation of two polarized waves in one aperture, which can expand the function of the LWIR camera and SAR-integrated imaging system. For example, dual-linearly polarized metasurface antennas can achieve full-duplex operation mode and be used for SAR imaging in HH, HV, VH and VV modes [16]. Functions such as cloud removal and image enhancement can be realized by using dual-linearly polarized metalens [17].

### 2.2. Dual-Polarized MIA Design

#### 2.2.1. Metasurface Principle

The reflector of the metasurface antenna is generally composed of periodic reflector units. In order to form the desired beam, the corresponding phase compensation of the units at different positions should be set reasonably. If the center of the reflector is the origin of coordinates, the coordinate system is established. According to the array antenna theory, when the beam direction is $\left(\theta_{0},\phi_{0}\right)$, the phase distribution of the reflector is

(1) $$\phi (x_{i},y_{j})=-\frac{2\pi }{\lambda_{S}}(\sin\theta_{0}\cos\phi_{0}x_{i}+\sin\theta_{0}\sin\phi_{0}y_{j})$$

where $\lambda_{S}$ is the wavelength of microwave in vacuum, and $\left(x_{i},y_{j}\right)$ is the central coordinate of the unit in row *i* and column *j*. With the phase center of the feed as the zero-phase reference point, then the electric field phase at each unit is equal to the sum of the path phase $\varphi_{t}$ and the transmission phase $\varphi_{b}$. As a result,

(2) $$\phi (x_{i},y_{j})=\varphi_{b}+\varphi_{t}=\varphi_{b}-\frac{2\pi }{\lambda_{S}}d_{ij}$$

where $d_{ij}$ represents the distance between the feed and the center of the unit, and the feed coordinates is $\left(x_{f},\;y_{f},\;z_{f}\right)$. According to Equations (1) and (2), the compensation phase of the metasurface antenna can be obtained as follows:

(3) $$\phi_{S}(x_{i},y_{j})=\frac{2\pi }{\lambda_{S}}(-\sin\theta_{0}\cos\phi_{0}x_{i}-\sin\theta_{0}\sin\phi_{0}y_{j}+\sqrt{{\left(x_{i}-x_{f}\right)}^{2}+{\left(y_{j}-y_{f}\right)}^{2}+{z_{f}}^{2}}).$$

When $\theta_{0}=0$, the above formula can be simplified to

(4) $$\phi_{S}(x_{i},y_{j})=\frac{2\pi }{\lambda_{S}}\sqrt{{\left(x_{i}-x_{f}\right)}^{2}+{\left(y_{j}-y_{f}\right)}^{2}+{z_{f}}^{2}}.$$

The metalens is also generally composed of periodic transmission units, and the incident light of the metalens is mostly vertical incidence; that is, $\theta_{0}=0$. So

(5) $$\varphi_{b}+\varphi_{t}=0=\varphi_{fb}+\varphi_{ft}$$

where, $\varphi_{t}$ and $\varphi_{b}$ are the path phase and transmission phase at the point $\left(x_{i}^{\prime },y_{i}^{\prime }\right)$, and $\varphi_{ft}$ and $\varphi_{fb}$ are the path phase and transmission phase at the center of the metalens, respectively.

For optical lenses, the focal length *f* is an important design parameter. In order to introduce *f* into the compensation phase formula, the zero-phase reference point of the transmission phase is taken at the center of the metalens; that is, $\varphi_{fb}=0$. So

(6) $$\varphi_{b}=\varphi_{ft}-\varphi_{t}=\frac{2\pi }{\lambda_{O}}(f-{\;d\;\prime }_{ij})$$

where $\lambda_{O}$ is the wavelength of the optical signal in vacuum, and $d_{ij}^{\prime }$ represents the distance between the focus and the center of the metalens unit. Therefore, the compensation phase of the metalens is as follows:

(7) $$\phi_{O}({x^{\prime }}_{i},{y^{\prime }}_{j})=-\frac{2\pi }{\lambda_{O}}(\sqrt{{{x^{\prime }}_{i}}^{2}+{{y^{\prime }}_{j}}^{2}+f^{2}}-f)$$

By analyzing the derivation process of the compensation phase of metasurface antenna and metalens, it can be found that the Equations (4) and (7) are equivalent. The difference between the two is as follows: First, the zero-phase reference point is different. Second, the location of the electromagnetic wave convergence is different.

Because the metasurface can flexibly control the polarization of electromagnetic wave, it can realize the convergence of two or more polarized wave with one aperture at the same time through reasonable design. When the focusing coordinates of the X- and Y-polarized light of the incident metalens are in the XOZ plane and symmetric on the Z axis, the compensation phase of the dual-linearly polarized metalens can be obtained by substituting the two focal points into the Equation (7):

(8) $$\phi_{x}(x_{i},y_{j})=-\frac{2\pi }{\lambda_{O}}(\sqrt{x_{i}^{2}+2x_{i}f\sin\alpha +y_{j}^{2}+f^{2}}-f)$$

(9) $$\phi_{y}(x_{i},y_{j})=-\frac{2\pi }{\lambda_{O}}(\sqrt{x_{i}^{2}-2x_{i}f\sin\alpha +y_{j}^{2}+f^{2}}-f)$$

where $\phi_{x}\left(x_{i},y_{j}\right)$ and $\phi_{y}\left(x_{i},y_{j}\right)$ represent the compensation phase of the X- and Y-polarized light at the point $\left(x_{i},y_{j}\right)$ on the metalens, respectively. And *f* is the focal length of the metalens, *α* is the off-axis angle of the focus. The focuses of the X- and Y-polarized light are $\left(-fsin\alpha ,0,-fcos\alpha \right)$ and $\left(fsin\alpha ,0,-fcos\alpha \right)$, respectively.

When the circularly polarized light passes through the metalens, the outgoing left-handed circularly polarized (LHCP) light and right-handed circularly polarized (RHCP) light are focused on the XOZ plane and are symmetric on the Z axis, and the compensation phase of the dual-circularly polarized metalens can be obtained:

(10) $$\phi_{LHCP}(x_{i},y_{j})=-\frac{2\pi }{\lambda_{O}}(\sqrt{x_{i}^{2}+2x_{i}f\sin\alpha +y_{j}^{2}+f^{2}}-f)$$

(11) $$\phi_{\mathrm{RH}CP}(x_{i},y_{j})=-\frac{2\pi }{\lambda_{O}}(\sqrt{x_{i}^{2}-2x_{i}f\sin\alpha +y_{j}^{2}+f^{2}}-f)$$

where $\phi_{LHCP}\left(x_{i},y_{j}\right)$ and $\phi_{RHCP}\left(x_{i},y_{j}\right)$ represent the compensated phase of LHCP and RHCP light at the point $\left(x_{i},y_{j}\right)$ on the metalens, respectively. The focuses of the LHCP and RHCP light are $\left(-fsin\alpha ,0,-fcos\alpha \right)$ and $\left(fsin\alpha ,0,-fcos\alpha \right)$, respectively.

#### 2.2.2. Design of Metasurface

The design steps of metasurface antenna and metalens can be divided into three steps: (1) design metasurface antenna and metalens unit; (2) establish the model of the metasurface antenna and metalens according to the compensation phase distribution; (3) conduct simulation and analyze the performance.

**Figure 2:**
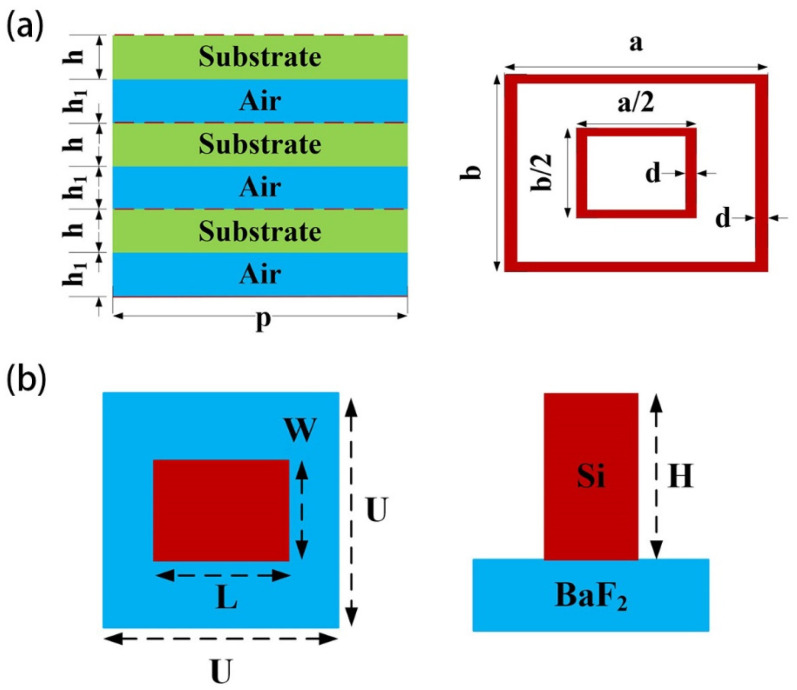
Schematic diagram of unit structure (**a**) metasurface antenna; (**b**) metalens.

The central wavelength of the metasurface antenna is 20 mm (15 GHz), the unit period is 10 mm, the unit shape is double rectangular ring, the dielectric substrate material is Rogers 5880 and the multi-layer structure is composed of three layers of dielectric plate and three layers of air layer, as shown in Figure 2a. In HFSS software (2022 R1), periodic boundary conditions are used to simulate the metasurface antenna unit.

The central wavelength of the metalens is 12 μm, the unit structure is Si nanorods (*n* = 3.411), the base material is BaF_2_ (*n* = 1.386), and the unit period is 6 μm, as shown in Figure 2b. In FDTD software (2020), periodic boundary conditions are used to simulate the metalens unit.

(1) Dual-linearly polarized metasurface unit

The key point of the design of dual-linearly polarized metasurface is to build a low loss unit library that can independently respond to two kinds of linearly polarized waves at the same time. The design of the unit library is as follows:

Firstly, the period, shape and size of the metasurface unit are determined. In order to realize the independent phase response to the two linearly polarized waves, the unit shape has at least two degrees of freedom, so the metasurface units are all rectangular. By changing the length and width of the rectangular unit at the same time, the transmission phase of the two polarized waves can be controlled from 0 to 360°. In order to meet the requirements of the phase range, the unit parameters need to be reasonably selected. The parameters of metasurface units in this paper are shown in Table 1.

**Figure 3:**
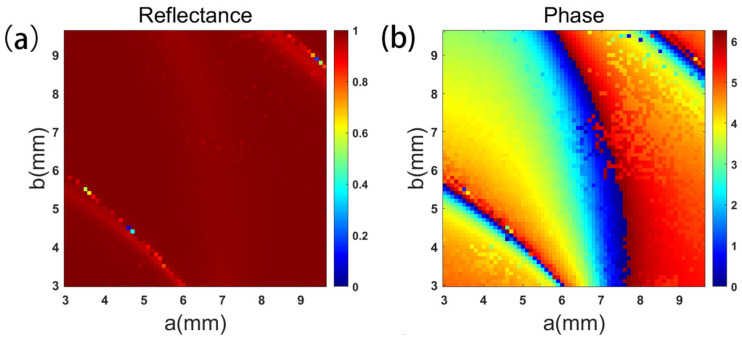
When the excitation is TE polarization, (**a**) the reflectance of the metasurface antenna units and (**b**) the transmission phase of the metasurface antenna units.

Then, the metasurface unit model is established and parameter scanning is performed. The excitation of the metasurface antenna unit model is set to TE and TM polarization in turn. In the range of 3~9.6 mm, the length and width of the double rectangular ring were changed, respectively, with the step size of 0.1 mm, and the changes in reflectance and transmission phase were recorded. The corresponding TE polarization results are shown in Figure 3. The source of the metalens unit model is set to X and Y polarized light in turn. In the range of 1~5 μm, the length and width of the rectangular nanorods were changed with the step size of 0.05 μm, and the changes of transmission phase and transmittance were recorded. The scanning results of the metalens were similar to those of the metasurface antenna.

Finally, the unit library is determined according to the different responses of the metasurface unit to two kinds of linearly polarized waves. If a linear polarized wave is set up with 8-order units, 64 different units are required for independent regulation of two linear polarized waves. In order to achieve good performance of the metasurface, a hierarchical screening method is used to find the units. First, under the condition of reflectance > 0.9 (or transmittance > 0.9 for metalens), the unit with the smallest absolute value of phase error with the target is screened. If the absolute value of the minimum phase error is greater than 32° or if there is no unit that meets the conditions, it is screened under the condition of reflectance > 0.85 (or transmittance > 0.85). The reflectance (or transmittance) threshold is then lowered appropriately until 64 phase units are determined. It is worth emphasizing that when the reflectance (or transmittance) threshold is <0.7, it is considered that the unit with good performance cannot be found. At this time, the parameters of the metasurface unit need to be properly adjusted, and then parameter scanning and unit screening of the adjusted unit are re-performed.

(2) Dual-circularly polarized metasurface unit.

Similar to the dual-linearly polarized metasurface unit, the design of the dual-circularly polarized metasurface unit library is as follows:

Firstly, the period, shape and size of the metasurface unit are determined. The unit parameters of the dual-circularly polarized metasurface are the same as those of the double linearly polarized metasurface.

Then, the phase response of the unit with respect to different circularly polarized waves is calculated. In order to simplify the design, the reflectance (or transmittance), transmission phase and conversion rate of LHCP and RHCP waves are calculated by using the scanning results of X- and Y-polarized waves. The conversion rate is defined as the proportion of the energy of the circularly polarized wave orthogonal to the incident wave to the total energy.

The method combining the transmission phase and P-B phase is used to regulate two orthogonal circularly polarized waves. When a circularly polarized wave passes through the metasurface unit, the reflected wave (or transmitted wave) can be represented by the Jones vector as follows [18]:

(12) $$\left[\begin{array}{l} E_{xout}\\ E_{yout} \end{array}\right]=T\frac{1}{\sqrt{2}}\left[\begin{array}{l} 1\\ ik \end{array}\right]=\frac{1}{2\sqrt{2}}\left\{(t_{u}+t_{v})\left[\begin{array}{l} 1\\ ik \end{array}\right]\right.+(t_{u}-t_{v})e^{-2ik\theta }\left.\left[\begin{array}{l} 1\\ -ik \end{array}\right]\right\}$$

where, $E_{xout}$ and $E_{yout}$ are the electric field components of reflected waves in the X and Y directions, respectively; *k* = ±1, corresponding to LHCP and RHCP waves, respectively; and *θ* is the rotation angle of the metasurface unit. Therefore, when the RHCP wave is incident, the output LHCP and RHCP waves can be obtained by the following:

(13) $$E_{LHCP}=E_{xout}-E_{yout};E_{RHCP}=E_{xout}+E_{yout}$$

where $E_{LHCPout}$ and $E_{RHCPout}$ are the outgoing electric fields of LHCP and RHCP waves, respectively.

Finally, the unit library is determined according to the different responses to the LHCP and RHCP waves. Assuming that $\varphi_{p}$ is the transmission phase of the metasurface unit, the relationship between $\varphi_{p}$, *θ* and the compensation phase is as follows:

(14) $$\varphi_{p}=\frac{\phi_{LHCP}+\phi_{RHCP}}{2};\theta =\frac{\phi_{LHCP}-\phi_{RHCP}}{4}.$$

**Figure 4:**
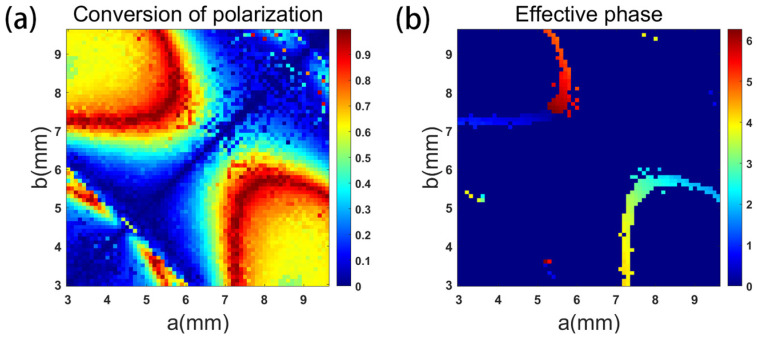
When RHCP wave is incident, (**a**) conversion rate of the metasurface antenna units and (**b**) effective transmission phase of the metasurface antenna units.

Among them, the transmission phase is realized by changing the length and width of the unit, and the P-B phase is realized by rotating the unit. Therefore, unlike the dual-linearly polarized metasurface, the dual-circularly polarized metasurface only needs to determine the 8-order unit library. In order to achieve good performance of the metasurface, hierarchical screening method is also used. Compared with dual-linearly polarized metasurface, the screening condition of dual-circularly polarized metasurface increases the conversion rate. Figure 4a represents the conversion rate of the metasurface antenna unit at the incidence of RHCP wave, and Figure 4b represents the transmission phase of the metasurface antenna with reflectance > 0.9 and a conversion rate of >0.9. According to the results of Figure 4b, the 8-order unit library of the metasurface antenna can be screened.

After the design of the metasurface unit, simulation models can be established in HFSS and FDTD, respectively, according to the compensation phase of the metasurface, and then the boundary conditions can be set up and the simulation calculation can be carried out.

## 3. Results and Discussion

### 3.1. Dual-Linearly Polarized MIA

The designed metasurface antenna is a reflection array of 20 × 20. The feed is a horn antenna with gain of 16.3 dB and 3 dB beamwidth of 27.4°. Two identical horn antennas operate in orthogonal polarization modes, and the feed coordinates are (0, −100, 173.2) and (0, 100, 173.2), in mm. The feed coordinates were substituted into the Equation (4) to obtain the phase distribution of the dual-linearly polarization metasurface, and the corresponding unit was selected from the unit library, then the simulation model of the metasurface antenna was established in HFSS. So the performance of the metasurface antenna can be obtained by calculation.

**Figure 5:**
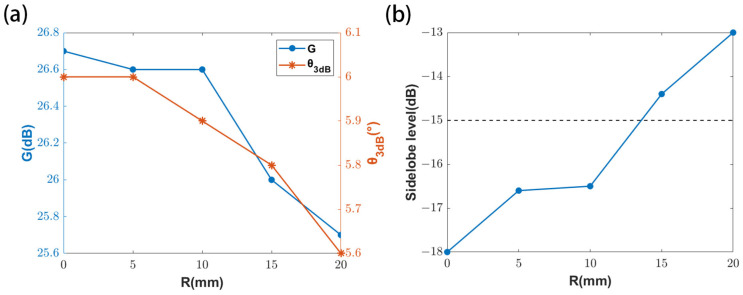
(**a**) The relationship between the gain and beamwidth of metasurface antenna and the radius of the metalens. (**b**) The relationship between sidelobe level of metasurface antenna and the radius of the metalens.

For the dual-polarized MIA, the metalens is embedded in the center of the metasurface antenna, so the electromagnetic compatibility is analyzed to determine the reasonable aperture of the metalens. The effect of metalens on the metasurface antenna is analyzed by opening a hole in the center of the metasurface antenna. When TE polarization is dominant, the relationship between the performance indicators of the metasurface antenna and the radius of the metalens are shown in Figure 5.

**Figure 6:**
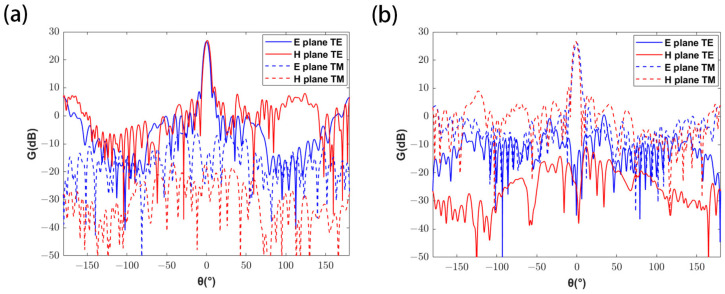
Patterns of the dual-linearly polarized metasurface antenna. (**a**) When TE polarization is dominant, antenna patterns on E and H planes. (**b**) When TM polarization is dominant, antenna patterns on E and H planes.

It can be seen from the above figure that, with the increase of the metalens radius, the gain and beamwidth of the metasurface antenna decrease, and the sidelobe level increases. According to Rayleigh’s criterion, a larger metalens radius can achieve higher resolution and obtain more energy; the metalens radius is determined to be 10 mm after considering the performance of the metasurface antenna and the metalens. At this time, the gain corresponding to the dual-linearly polarized metasurface antenna is 26.6 dB and 26.0 dB, respectively, with the 3 dB beamwidths of 5.9° and 6.2°, and the sidelobe levels of −16.5 dB and −16.3 dB, respectively. And the antenna patterns are shown in Figure 6.

**Figure 7:**
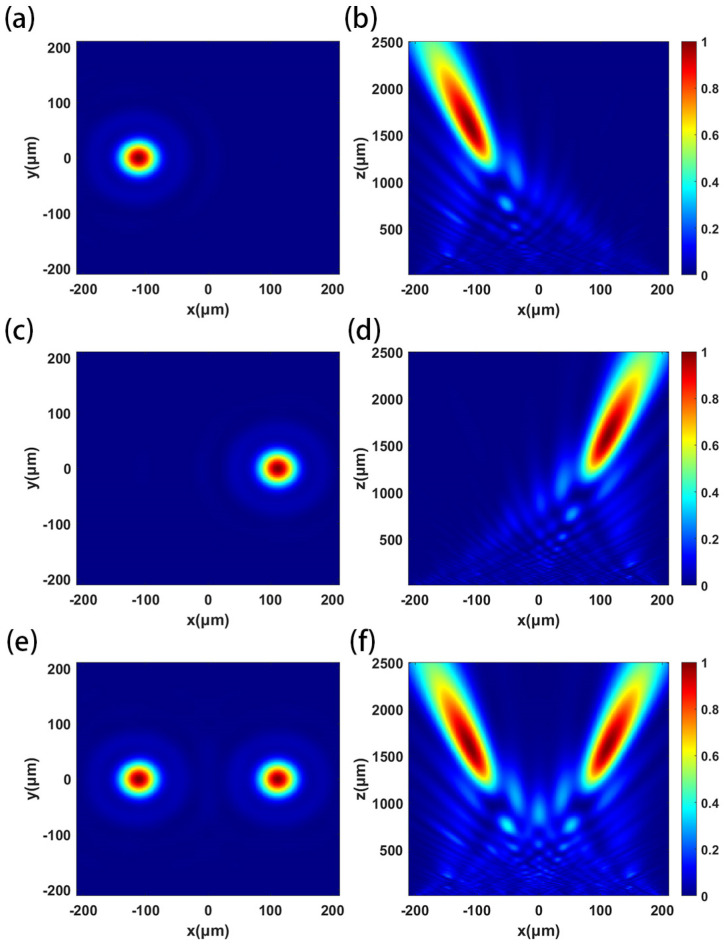
Normalized distribution of the square of the electric field intensity when the X-polarized light, Y-polarized light and 45°-polarized light are incident in turn: (**a**,**c**,**e**) XOY plane; (**b**,**d**,**f**) XOZ plane.

The designed metalens has a diameter of 20 mm and a focal length of 100 mm. Because the period of the metalens is 6 μm, the number of units is about 8.7 × 10^6^, which cannot be simulated by an ordinary computer. Therefore, the same scale reduction model is used to simulate and analyze the metalens. Keeping the relative aperture of the metalens unchanged, the simulated metalens diameter is 420 μm, the focal length is 2100 μm, and the off-axis angle α of the focus is 4°. The phase distribution can be calculated according to the compensation phase of the metalens. The metalens source is set to X-polarized light, Y-polarized light and 45°-polarized light in turn, and the focusing effect is shown in Figure 7. The actual focal length of the metalens is 1604 μm, and the focusing efficiency of X-polarized light and Y-polarized light is 63.6% and 64.1%, respectively. The extinction ratio of the two orthogonal polarized lights is 0.03 dB.

### 3.2. Dual-Circularly Polarized MIA

**Figure 8:**
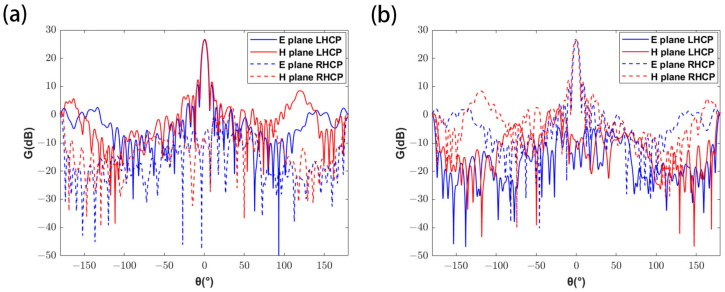
Patterns of dual-circularly polarized metasurface antenna. (**a**) When LHCP is dominant, antenna patterns on E and H planes. (**b**) When RHCP is dominant, antenna patterns on E and H planes.

The dual-circularly polarized metasurface antenna is also designed as a 20 × 20 array. Two identical circular horn antennas operate in RHCP and LHCP modes, respectively. And their corresponding coordinates are (0, −100, 173.2) and (0, 100, 173.2), in mm. The feed antenna has gain of 11.9 dB and 3 dB beamwidth of 50.2°. The corresponding unit was selected from the unit library, and the simulation model of the metasurface antenna was established in HFSS. A hole with a radius of 10 mm was cut in the center of the model. After calculation, the patterns corresponding to the double polarizations are shown in Figure 8. The gain of the dual-circularly polarized metasurface antenna is 26.6 dB and 26.6 dB, respectively, with the 3-dB beamwidths of 5.5° and 5.6°, and the sidelobe levels of −14.4 dB and −14.3 dB, respectively.

**Figure 9:**
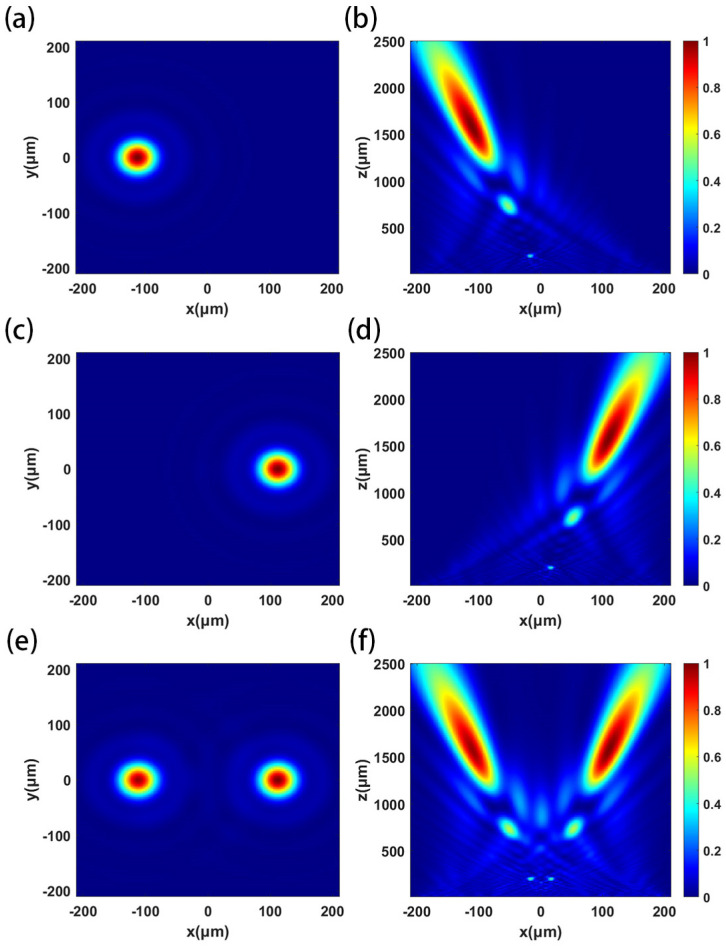
Normalized square distribution of electric field intensity when RHCP light, LHCP light and 45°-polarized light are incident in turn: (**a**,**c**,**e**) XOY plane; (**b**,**d**,**f**) XOZ plane.

The simulated dual-circularly polarization metalens has a diameter of 420 μm, a focal length of 2100 μm, and the off-axis angle α of the focus is 4°. The phase of the metalens at different positions is calculated to establish the metalens model. The light source is set to RHCP light, LHCP light and 45°-polarized light in turn, and the focusing effect is shown in Figure 9. The actual focal length of the metalens is 1604 μm, and the focusing efficiencies of RHCP and LHCP light are both 65%. The extinction ratio of the orthogonal circularly polarized lights is 0.07 dB.

## 4. Conclusions

In this paper, a dual-polarized MIA for LWIR cameras and SAR-integrated imaging was designed to solve the problems of complex structure, large volume and unfavorable integration. It is composed of a microwave metasurface antenna and an optical metalens, and the metalens was embedded in the center of the metasurface antenna. The MIA utilized the powerful electromagnetic wave control ability and its thin structure, which significantly simplified the optical and microwave signal transmission paths and made the system structure simpler and easier to integrate. The dual-linearly polarized and dual-circularly polarized MIAs were designed and simulated. The results show that the dual-polarized MIA is reasonable in design. This paper provides a new scheme for LWIR cameras and SAR-integrated imaging systems with simple structure, diverse functions and easy integration, which can promote the development of multi-sensor integration technology.

## Figures and Tables

**Table 1:** Parameters of metasurface units.

Metalens	Parameters	L/μm	W/μm	H/μm	U/μm		
	Value	[1,5]	[1,5]	8	6		
Antenna	Parameters	a/mm	b/mm	d/mm	h/mm	h_1_/mm	p/mm
	Value	[3,9.6]	[3,9.6]	0.3	1	1	10

## Data Availability

The data presented in this study are available from the corresponding author upon reasonable request.
